# Usability Evaluation Methods Used to Ensure the Usability of the MEDSReM-2 System

**DOI:** 10.1093/geroni/igab046.884

**Published:** 2021-12-17

**Authors:** Timothy Hale, Carson Smith, Mimi Trinh, Wendy Rogers

**Affiliations:** 1 University of Illinois Urbana-Champaign, University of Illinois Urbana-Champaign, Illinois, United States; 2 University of Illinois Urbana-Champaign, Champaign, Illinois, United States

## Abstract

A key objective of the MEDSReM-2 study is to promote medication taking decisions and improve adherence to hypertensive medications for older adults. New functionalities include enhanced decision-support algorithms for missed medications, automated entry of blood pressure measurements, improved data visualizations, and an easy-to-use online web portal. In support of these enhancements, the User Testing subteam is tasked with providing ongoing evaluation and feedback on the usability of early design concepts, prototypes, beta software, wireless blood pressure monitors, and instructional materials. The overall project comprises multiple working teams, whose efforts must be coordinated. We will describe the challenges of working with these interdisciplinary teams and the usability evaluation methods used to support the needs of each team in creating the enhanced MEDSReM-2 system that is easy-to-use and effective in helping older adults improve their hypertension medication adherence. These processes inform the research and design efforts of other technology interventions.

